# Multibeam bathymetry data from the Kane Gap and south-eastern part of the Canary Basin (Eastern tropical Atlantic)

**DOI:** 10.1016/j.dib.2020.106055

**Published:** 2020-07-24

**Authors:** Ivan Yu. Dudkov, Vadim V. Sivkov, Dmitry V. Dorokhov, Leyla D. Bashirova

**Affiliations:** a Shirshov Institute of Oceanology, Russian Academy of Sciences, 36, Nahimovskiy prospekt, Moscow, Russia; b Immanuel Kant Baltic Federal University, Nevskogo Street, 14, Kaliningrad, Kaliningrad Region 236041, Russia

**Keywords:** Multibeam bathymetry, Digital elevation model, Deep ocean gateway, Geomorphology, Eastern tropical Atlantic, Kane Gap, Canary Basin, Gambia Basin

## Abstract

We present a multibeam bathymetry dataset from eastern part of the Canary Basin and from the Kane Gap located north and south of the Cape Verde Islands, respectively. Special attention was paid to the deep-water Kane Gap between Sierra Leone Rise and Guinea Plateau. The Kane Gap is the only possible gateway that allows the exchange of Antarctic Bottom Water between the Sierra Leone and Gambia Basins. Bathymetry surveys were carried out during the 44th cruise of the Research Vessel (R/V) “Akademik Nikolaj Strakhov” in October 2019. The data were collected using the system RESON SeaBat 7150 (12.5 kHz) and processed using QINSy software. The multibeam bathymetry data are presented in tabular format (ASCII (*.txt)) and as digital elevation models in ESRI ASCII grid (*.asc) and GeoTIFF raster (*.tif) formats with a resolution of 100 m. The dataset is available with the article.

Specifications Table

**Table:** 

**Subject**	Seafloor geomorphology
**Specific subject area**	Multibeam bathymetry
**Type of data**	Tabular data, digital elevation models (DEMs)
**How data were acquired**	Field survey, shipboard acquisition system. Multibeam echosounder RESON Seabat 7150, frequency 12.5 kHz.
**Data format**	Raw and analysed: Tabular data: ASCII table (*.txt), DEMs: ESRI ASCII grid (*.asc), GeoTIFF raster (*.tif)
**Parameters for data collection**	Vessel speed 6–8 knots during multibeam survey. The survey was designed as a series of parallel swaths with a 10–40% (up to 60–75%) overlap.
**Description of data collection**	The raw multibeam data were processed using QINSy software (QPS, Netherlands). Validated multibeam data were converted into the ASCII-table (*.txt).The DEMs were created by triangle surface method and converted into ESRI ASCII grid (*.asc) and GeoTIFF raster (*.tif) in QINSy and ArcGIS software.
**Data source location**	Shirshov Institute of Oceanology, Russian Academy of Sciences, Kaliningrad, Russia
**Data accessibility**	Data are presented with this article

**Value of the Data**

- Up to date, bottom topography of the abyssal ocean plains and deep-sea channels and passages controlling the deep water exchange between the ocean basins in the East Atlantic is still poorly studied. The developed digital elevation models (DEMs) represent a high-detailed bathymetry of the Canary Basin and the Kane Gap. The data obtained significantly contribute to our knowledge about sedimentary and hydrological processes in the ocean gateways.
- For the first time, an expressive structure of the giant deltaic contourite fan has been revealed north of the Kane Gap. Previously, only a small part of this fan has been reported and described [1]. In the Canary Basin, such bathymetric features as distal parts of the submarine canyon and a separate elongated hill in the Antarctic Bottom Water (AABW) affected area have been revealed by the new DEM.
- The data are useful for hydrologists, sedimentologists, paleoceanographists, and geomorphologists who study the AABW modern and past circulation, contourites, and their formation.
- The data advance our knowledge about the spreading of the AABW in the East Atlantic and provide the information for the paleoceanographic study.
- The data are valuable for planning the research on the local polygons with mooring sites for the current velocity registration and for the planning of the sediment cores sampling taking into account the bottom topography.
- The data about the AABW modern and past circulation can serve the information about global climate changes, as it is the coldest water in the World Ocean and any change in the properties and routes of AABW (e.g. increase of the temperature) can have consequences for the global climate.
- The data about the formation of the contourite drifts are of high value as the contourites are the potential reservoirs for hydrocarbons.
- The data can be contributed to help update the GEBCO database and can be used in educational programs.

## 1 Data description

All the data are presented as the supplementary data to the article.

The dataset includes:

- Two ASCII text files (*.txt) of processed bathymetry data collected by multibeam echosounder Reson SeaBat 7150 (the structure is presented in Table 1);

**Table 1:** Description of columns in ASCII text table.

Column Name	Description
System	Multibeam echosounder system
Date	Date of survey, yyyymmdd.
Time	Time of survey (UTC), hhmmss.
Easting	Easting Cartesian coordinate, UTM Zone 27 N projection, WGS84.
Northing	Northing Cartesian coordinate, UTM Zone 27 N projection, WGS84.
Depth	Corrected and processed depth, m.
Latitude	Geographical latitude, DD;MM.mm, WGS1984.
Longitude	Geographical longitude, DD;MM.mm, WGS1984.
- Two ESRI ASCII grids (*.asc) DEMs with a scale 1:100 000, spatial resolution of 100 m in the UTM Zone 27 N projection, WGS84;
- Two GeoTiff rasters (*.tif) of filtered DEMs (low pass 3-by-3 filter) with a scale of 1:100 000, spatial resolution of 100 m in the UTM Zone 27 N projection, WGS84.
- Three figures (*.png). Fig. 1 shows the investigated area, Fig. 2, Fig. 3 show the developed DEMs.

**Fig. 1:**
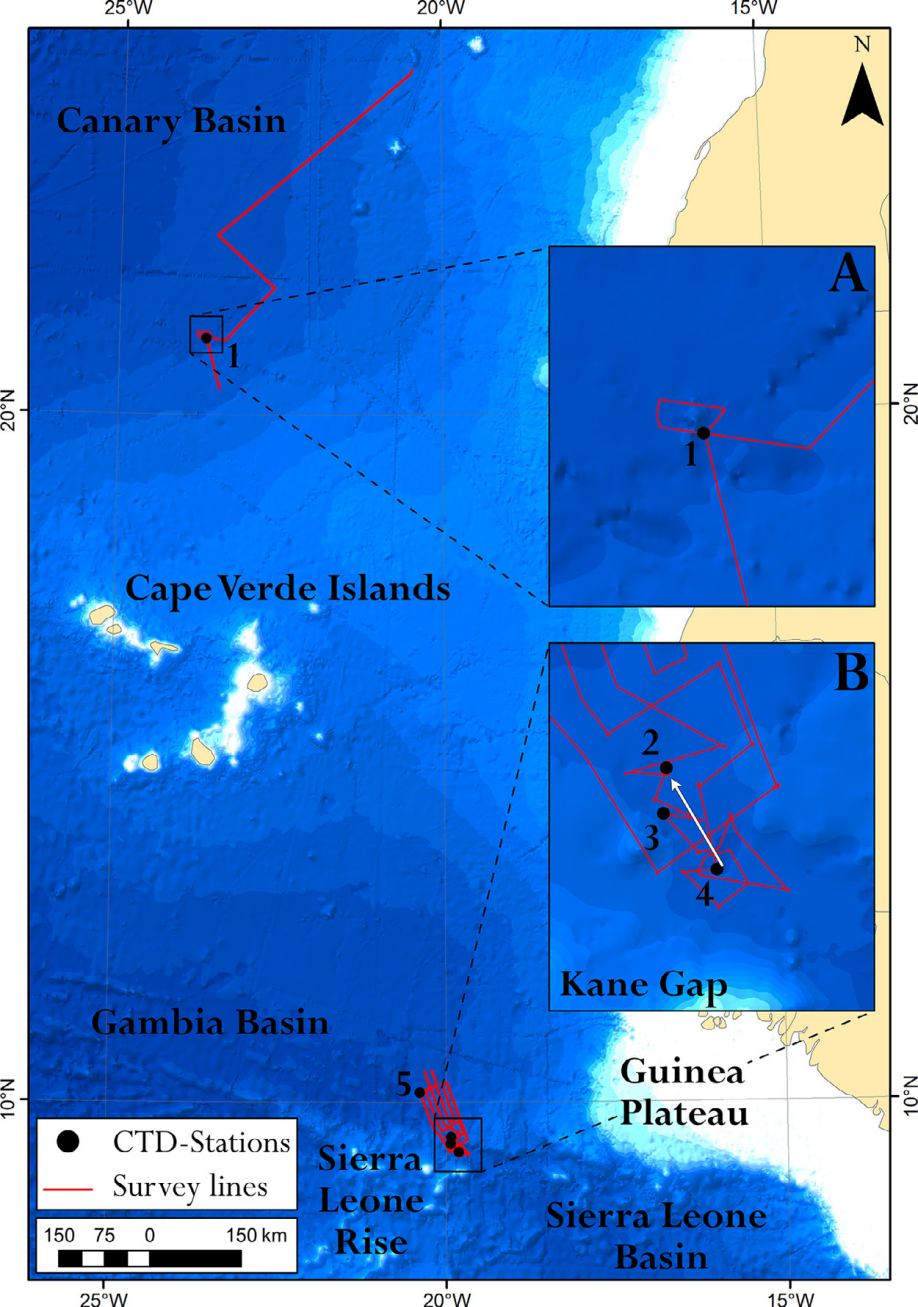
Areas investigated during the 44th cruise of the R/V “Akademik Nikolaj Strakhov”: A – polygon in the south-eastern slope of the Canary Basin; B – polygon in the Kane Gap area. Black circles mark the CTD-Stations of R/V “Akademik Nikolaj Strakhov”: 1 –ANS-44,001; 2 – ANS-44,003; 3 – ANS-44,004; 4 – ANS-44,006; 5 – ANS-44,007. The white arrow on inset map B shows the dominant direction of the bottom current of the AABW. Bathymetry is based on the SRTM 15 arc-second global relief [3] (shown with blue color).

**Fig. 2:**
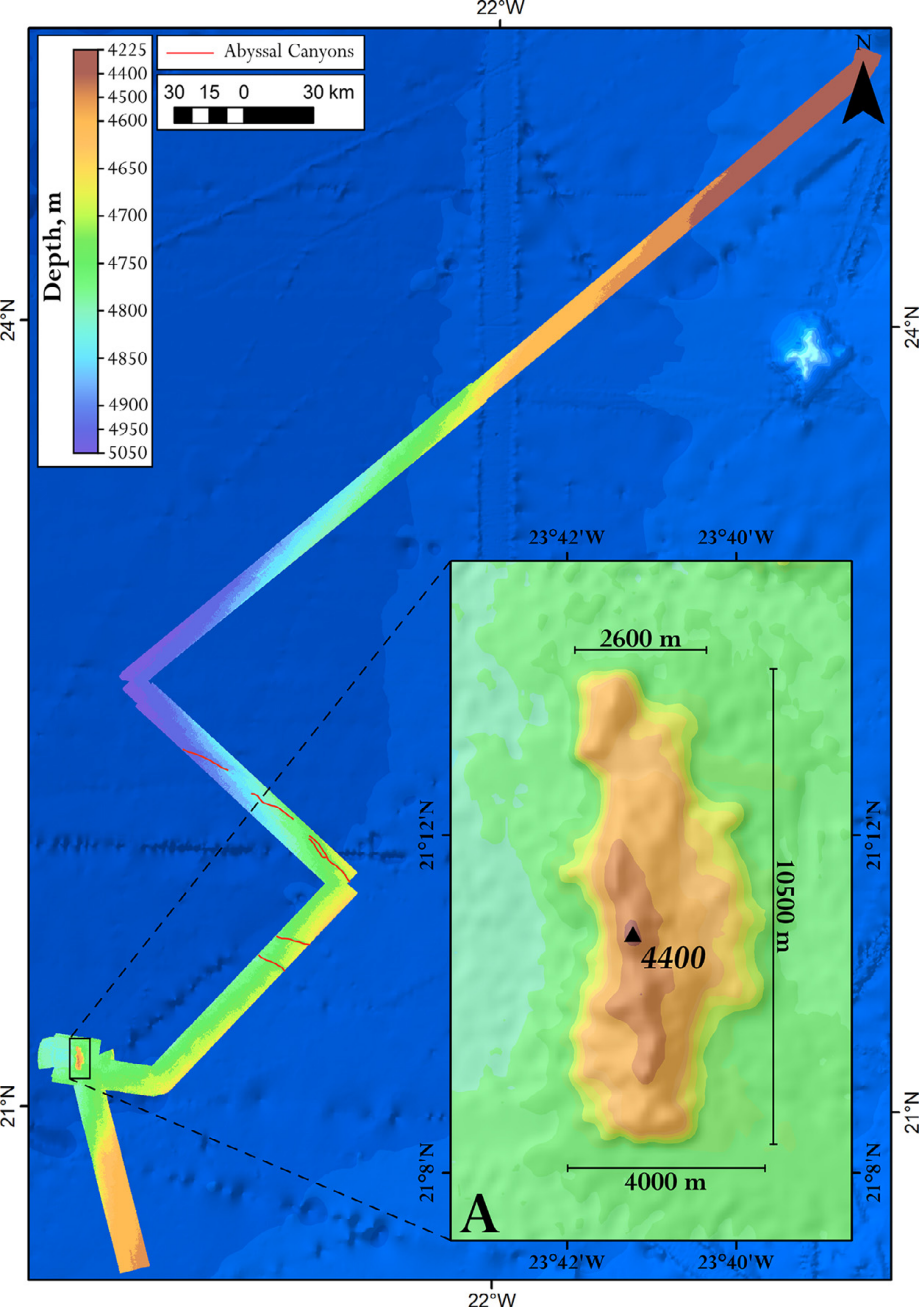
General view of the bathymetric data (DEM) of the south-eastern slope of the Canary Basin received during the 44th cruise of the R/V “Akademik Nikolaj Strakhov”. Distal parts of submarine canyon are given by red lines. Map inset (A) shows a separate elongated (volcanogenic?) hill. Bathymetry is based on the SRTM 15 arc-second global relief [3] (shown with blue color).

**Fig. 3:**
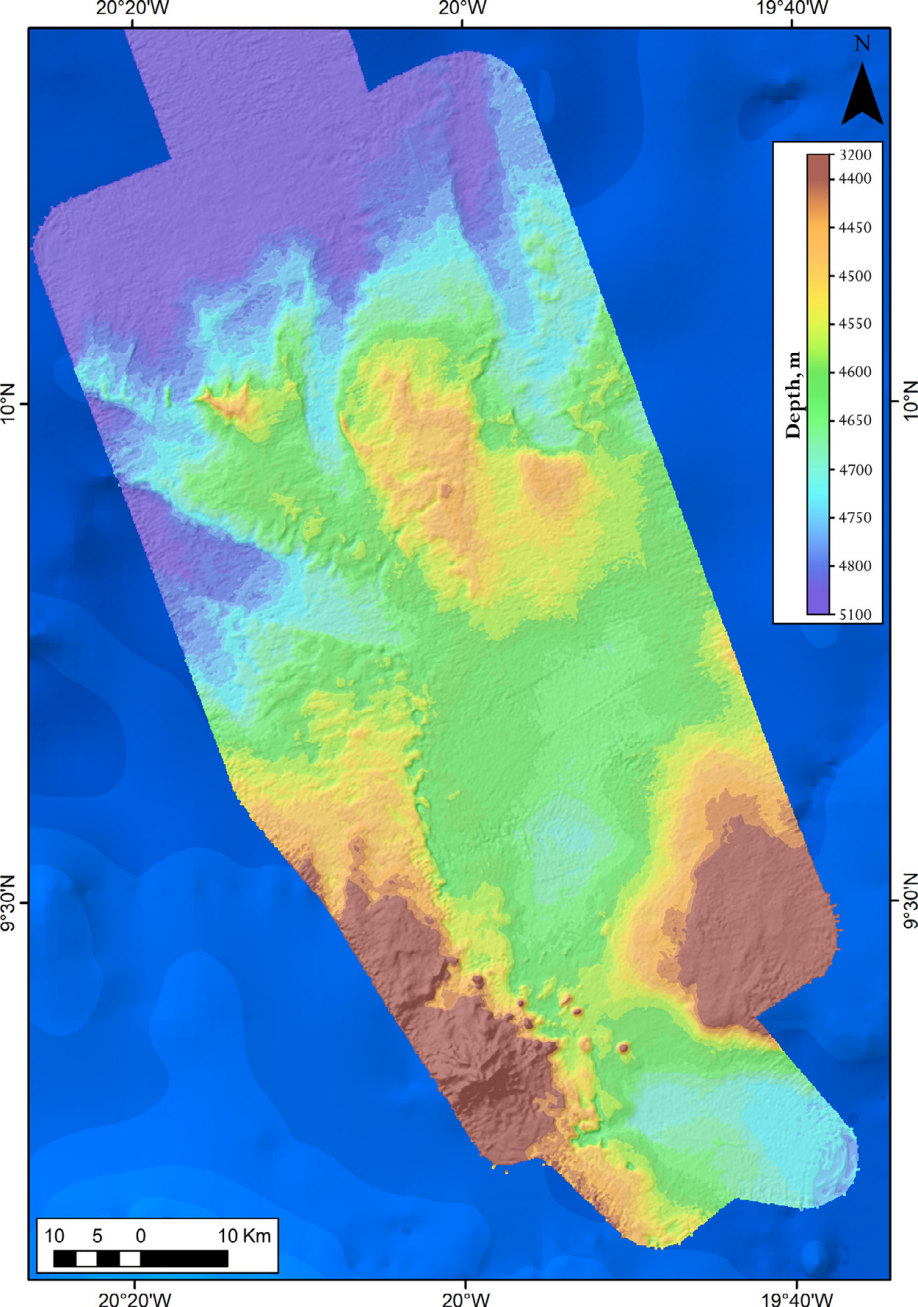
Bathymetry (DEM) of the Kane Gap area received during the 44th cruise of the R/V “Akademik Nikolaj Strakhov”.

Provided data are fully processed.

The investigated area is shown on Fig. 1. The developed DEMs are provided on Fig. 2 and 3.

## 2 Experimental design, materials, and methods

High-resolution multibeam bathymetry (MBE) surveys were carried out in the Canary Basin and in the Kane Gap area during the 44th cruise of the R/V “Akademik Nikolaj Strakhov” in October 2019 (Fig. 1). Single swath of MBE covers 8–10 km. The distance between measured depths is 70–100 m along and ∼80 m across the survey line. The spatial resolution of DEM is 100 × 100 m. The total length of survey lines is 1320 nautical miles.

The survey was designed as parallel swaths with a 10–40% (up to 60–75%) overlap, and the vessel speed was 6–8 knots. The data were collected using the system RESON SeaBat 7150 (12.5 kHz) with an integrated navigation system Applanix POS MV. For data processing QINSy software was applied. Current and wave effects were corrected by motion-control sensor of Applanix POS MV. Tidal corrections were ignored.

To correct the multibeam data during the post-processing stage, sound velocities in the water column (SVP-correcion) were calculated from temperature, salinity, and pressure data from the SBE19v2 Plus CTD. The CTD measurements were carried out at the oceanographic stations shown in Fig. 1. For SVP-corrections we used two modules of the QINSy software – “Sound Velocity Processor” and “Refract filter”. The first module allowed adjusting the measured depths using a mean sound velocity in the water column from the CTD-profile. The second module corrects the data for beam refraction. Parameters of refraction layers were detected using the sound velocity profiles.

Data post-processing, data processing in QINSy, and developing the DEMs are fully described in [2]. The resulting bathymetry is presented in UTM 27 zone, datum WGS84 (Figs. 2 and 3).

## Declaration of Competing Interest

The authors declare that they have no known competing financial interests or personal relationships which have, or could be perceived to have, influenced the work reported in this article.

## Appendix Supplementary materials

Image, application 1

Image, application 2
